# Application Layer ARQ Algorithm for Real-Time Multi-Source Data Streaming in UAV Networks

**DOI:** 10.3390/s21175763

**Published:** 2021-08-27

**Authors:** Mohammed Amin Lamri, Albert Abilov, Danil Vasiliev, Irina Kaisina, Anatoli Nistyuk

**Affiliations:** Department of Networks and Telecommunication Systems, Faculty of Instrumental Engineering, Kalashnikov Izhevsk State Technical University, ul. Studencheskaya, 7, 426069 Izhevsk, Russia; albert.abilov@istu.ru (A.A.); danil.s.vasiliev@gmail.com (D.V.); irinakaysina25@gmail.com (I.K.); nai@udm.ru (A.N.)

**Keywords:** buffer management, UAVs, real-time application, delay, packet loss rate, jitter, quality of service

## Abstract

Because of the specific characteristics of Unmanned Aerial Vehicle (UAV) networks and real-time applications, the trade-off between delay and reliability imposes problems for streaming video. Buffer management and drop packets policies play a critical role in the final quality of the video received by the end station. In this paper, we present a reactive buffer management algorithm, called Multi-Source Application Layer Automatic Repeat Request (MS-AL-ARQ), for a real-time non-interactive video streaming system installed on a standalone UAV network. This algorithm implements a selective-repeat ARQ model for a multi-source download scenario using a shared buffer for packet reordering, packet recovery, and measurement of Quality of Service (QoS) metrics (packet loss rate, delay and, delay jitter). The proposed algorithm MS-AL-ARQ will be injected on the application layer to alleviate packet loss due to wireless interference and collision while the destination node (base station) receives video data in real-time from different transmitters at the same time. Moreover, it will identify and detect packet loss events for each data flow and send Negative-Acknowledgments (NACKs) if packets were lost. Additionally, the one-way packet delay, jitter, and packet loss ratio will be calculated for each data flow to investigate the performances of the algorithm for different numbers of nodes under different network conditions. We show that the presented algorithm improves the QoS of the video data received under the worst network connection conditions. Furthermore, some congestion issues during deep analyses of the algorithm’s performances have been identified and explained.

## 1. Introduction

At present, the transport protocols of the Open System Interconnection (OSI) model, Transmission Control Protocol (TCP) and User Datagram Protocol (UDP), are facing a lot of challenges in real-time applications over UAV networks due to specific characteristics such as their time-sensitive nature to the high mobility of nodes. When a real-time multi-source download scenario is applied, the multiple paths to the same receiving node increase interflow interference, cause network congestion, reduce the performance’s gain, and lead to packet loss. Packet loss will affect the QoS of the application’s stream [1], regardless of which transmission protocol is used.

To monitor and provide the highest (QoS) of the network and alleviate packet loss, a good error control strategy must be implemented depending on the scenario proposed. This strategy must handle the interference imposed by the multiple paths, keep the packet loss ratio and the application delay under the required limits and boost the throughput and network reliability. The most convenient solution for such a problem is to develop a custom ARQ mechanism with a shared buffer on the application layer, where only the lost packets are selectively retransmitted. The main QoS key parameters that should be taken into consideration are packet loss rate and transmission delay [2]. According to [3], there are three principal causes behind the impairment of video quality during the wireless transmission process:

- Distortions caused by digital compression;
- Distortion during the analog to digital conversion process;
- Distortion due to error-prone transmission.

Because our algorithm is based on a reactive end-to-end QoS control strategy, distortion due to error-prone transmission including signal attenuation, competition for media access and buffer issue is considered as the main cause of impairment of video quality.

Much research has been carried out for multi-source data transmission for different scenarios. As an example, the authors in [4] proposed a distributed joint source, routing, and channel selection scheme for a cooperative multi-source network, where a set of transmitters send parts of the same data file to the requested node. For the network layer, the authors in [5] proposed an information-centric networking, where in-network caching can be collaborated with network coding to achieve multi-source transmission. The authors in [6] proposed a combined cross-layer design of adaptive modulation and coding at the physical layer with a truncated ARQ protocol at the data link layer, in order to maximize spectral efficiency under prescribed delay and error performance constraints. This work was analyzed and improved in [7] by designing a new rule of Adaptive Modulation and Coding (AMC), which is used to adaptively choose the transmission mode. In [8], the authors combine cross-layer error protection techniques composed of an error correction code in the link/MAC layer, erasure code in the application layer, and ARQ across the link/MAC layer and application layer. While these works show huge potential for multi-source download, they are based on simulation results and lack a rigorous analytical model and a good combination of ARQ mechanism and a multi-source download scenario.

To this end, the motivation of this work is the development of an application algorithm, ARQ, for a multi-source one-sink download scenario with a shared receiving buffer to manage packet reordering and packet recovery. The algorithm proposed is based on the research carried out by authors in [9].

Since the customization of radio interfaces such as WiFi, 4G, and 5G is often an expensive task, to improve the QoS in a real deployed scenario by developers of UAVs networks, the most acceptable option at minimal cost is to use recovering methods of lost data fragments programmatically on the application layer. Such methods with Application Layer ARQ purposes are currently sufficiently studied, but in most cases, scenarios with one transmitter and one receiver are considered. Our main contribution is to improve the QoS of real-time multi-source data transmission and provide the basis for measuring QoS metrics through the use of a new error control algorithm based on MS-AL-ARQ.

The remainder of this article is organized as follows. Section 2 explains the conception of the proposed algorithm. Section 3 presents the QoS measurement metrics proposed for the algorithm. Section 4 reports the comprehensive experimental results. Section 5 concludes the paper.

## 2. Multi-Source Streaming Algorithm MS-AL-ARQ

The algorithm proposed is designed to run over UDP. The reason behind this choice is that for other reliable transmission protocols such as TCP, retransmission of old and probably expired packets will affect the QoS of the application’s stream and increase the application’s delay. Moreover, since UDP applications are connectionless, they can accept several concurrent data flows from different transmitters. However, for each flow, the data processing and the identification of the sender must be carried out in the application layer to achieve a reliable transmission.

Our mechanism, MS-AL-ARQ, is designed to listen to data packets coming from several sources on the same socket (IP/port) using a single receiver thread. The reason for choosing a single receiving thread and not the multi-threading concept is due to several reasons, such as the high complexity of the multi-threading coding and the difficulty to handle concurrency [10]. Additionally, the identification and correction of errors are much more difficult in multi-threading processes compared to single-threaded processes [11].

**Figure 1:**
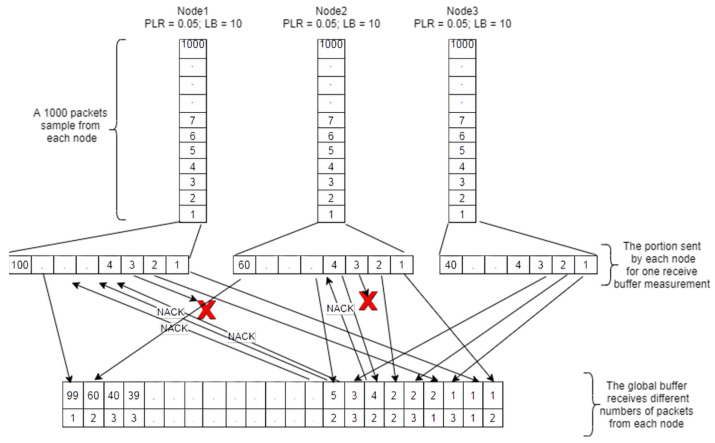
Multi-source data transmission schema for MS-AL-ARQ. PLR: Packet Loss Ratio; LB: Lost Burst.

We focus on multi-source transmission, where a set of transmitters *N* sends data to the same receiver; each transmitter has its own channel and its own source data. Figure 1 shows the global buffer content in the case of three senders. The first row represents the sequence number of each packet and the second row represents the identification number of the sender. As shown in the example, the buffer detects a loss event on the data flow of the second node (received from node 2, packet number 2, then packet number 4). In this case, and after the packet has been stored in the buffer, the function *send_NACK ()* will be invoked to send the *NACK* packet. As a sequence, the retransmission number (*RN*) included in the packet’s header will be incremented for each request.

**Figure 2:**
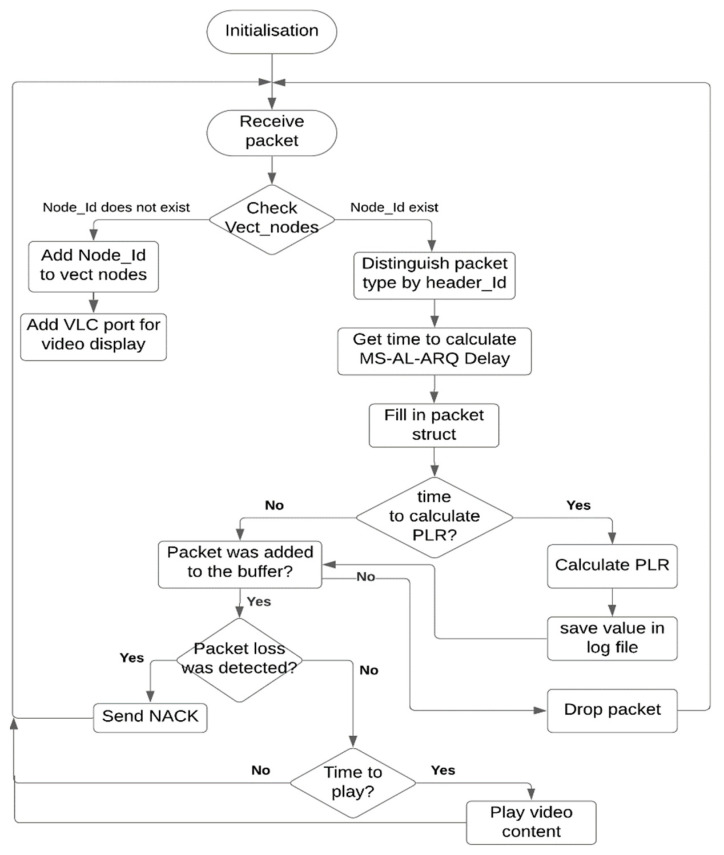
MS-AL-ARQ flow diagram.

Figure 2 represents the flow diagram of the MS-AL-ARQ algorithm (client side) on the application layer where the distinguishing, processing, and displaying of video data are handled. The algorithm uses global buffer to store all the packets received from the transmitters. This buffer will handle loss events, packet retransmission, and packet duplication that occurred during the transmission of packets from each node using a combination of the packet’s sequence number and the sender’s identification number (*SN; Node_Id*). If a loss event was detected, a *NACK* will be formed as a combination of its repeat number, sequence number (*SN*) and the amount of burst. The *NACK* packet will be sent to the appropriate node according to its identification number *Node_Id* (which is a string type value containing the IP address of the sending node).

The data-carrying packets received from the different nodes will be distinguished based on their identification number (*ID*); if the *ID* = 7, the client application will tag the received packet as a data packet (*ID* = 8 for the retransmitted packet, *ID* = 12 for ping packets, *ID* = 13 for cancellation messages). Thereafter, only the data-carrying packets will be stored in the global buffer in ascending order depending on their sequence number *SN* and the node’s identification number *Node_Id*. According to these parameters, the packet loss event will be detected for each node and the *NACK* packet will be formed. The global buffer will also detect the duplicated packets received in the case where the *NACK* packet was sent as a request for a sequence of lost packets but not all of them were received from the first retransmission. This case is particularly related to the retransmission time out (RTO) and round trip time (RTT) values calculated during the transmission [9]. In this case, the buffer will drop the duplicated packets.

## 3. Quality of Service Metrics Measurement

### 3.1. Packet Loss Rate

In wireless networks, packet loss events occur when one or more packets fail to reach their destination [12]. This will affect the QoS over the network as well as the quality of experience (QoE). In real-time applications such as video streaming over UAV networks, packet loss tends to be very high because of the unpredictable changes in network topology and application requirements [13]. In this case, the data flow must be guaranteed at certain data delivery rates for smooth and seamless transmission, and the number of packets lost or dropped during transmission must be kept low. In a transmission interval (1000 packets, in our case), the packet loss rate (PLR) can be calculated as follows:

(1) $$\mathrm{PLR}=\frac{N^{tx}-N^{rx}}{N^{tx}}\cdot 100\%$$

where $N^{tx}$ is the number of generated and transmitted packets and $N^{rx}$ is the number of received packets.

**Figure 3:**
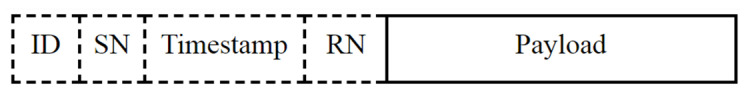
Data packet format. ID: identification of the packet type; SN: sequence number; RN: number of retransmission.

This evaluation can be easily performed by extracting the number of received packets from the amount of sent ones. However, since the calculation of PLR will be performed on the destination node for each data flow, it was necessary to calculate the amount of packets transmitted by the sender using the assigned SN in the packet’s header (Figure 3).

Algorithm 1 shows the pseudo-code of the packet loss rate calculation function for MS-AL-ARQ.
**Algorithm 1.** Packet loss rate calculation for MS-AL-ARQ.1.**procedure** PlrCalculation(Node_id, SN)>Initialization2.Create plr_vect for the node
3.**if** plr_vect.empty() = True **then**
4.  prev = SN
5.  plr_vect.Add(prev)
6.**Else**
7.  curr = SN
8.  global_ct ++>counter of received packets9.  **if** curr ≠ (prev + 1) && (curr ≠ prev) **then**
10.    **if** (curr - prev - 1 > 0 ) && (curr **is not in** plr_vect) **then**
11.     plr_vect.Add(curr)
12.     lost_ct += (curr - prev - 1)>counter of lost packets13.     prev = curr
14.  **else if** curr < prev **then**
15.    **if** curr **is not in** plr_vect **then**
16.     plr_vect.Add(curr)
17.     rec_ct += 1>counter of recovered packets18.
  **Else**

19.    prev = curr


During the experimentation phase, we defined the number of sending nodes statically. This will define the size of the vector *vect_node*, which contains the Identification numbers *Node_id* of each sender. These identification numbers will be used to execute the convenient instance of the function *PLR_calculate()* to calculate the packet loss rate of the related node. After the initialization of all the variables, the function will check the vector of sequence numbers *plr_vect* if it is empty. If true, the sequence number of the received packet will be marked as previous (*prev*); then, it will be added in the vector of sequence numbers *plr_vect*. If false, then the SN will be marked as (*curr*). Thereafter, a sequence of tests will be executed to check whether this packet is a new packet, a requested packet, or a duplicated one. Accordingly, a counter for each type of packet will be increased to determine the total of packets received and retransmitted.

### 3.2. Delay and Delay Jitter

For a given flow $f_{n}$, the total number of packets $P_{n}$ received and reordered in the buffer will be determined by the two main reordering metrics: reordering byte offset and reordering late time offset. Both metrics will be set by the global buffer B(r,t), where *r* and *t* represent reordering byte offset and reordering late time, respectively. The algorithm proposed for one-way packet delay calculation requires registered timestamps (sending time) of each transmitted packet. These timestamps will be included in the packet header formed by the application. From this context, for each packet $P_{i}$ ∈ $f_{n}$ in B(r,t), the one-way delay $d_{i}$ will be measured as:

(2) $$d_{i}=t_{i,reception}-t_{i,transmission}$$

where $t_{i,reception}$ and $t_{i,transmission}$ are, respectively, the reception time and transmission time of the packet $P_{i}$.

For accurate packet delay measurement, time synchronization between the sender system and the receiver system must be provided. Therefore, the network time synchronization protocol (NTP) has been used to provide a frame of time reference between the sender and receiver [14]. The proposed function includes the following features:

1. The NTP offset (known as the difference in time between an external timing reference and time on a local machine) can affect the accuracy of delay measurement. If the NTP offset is higher than a predefined threshold (0.5 ms), the $t_{i,transmission}$ value will be adjusted as follows:
  (3) $$t_{i,transmission}=t_{i,transmission}-NTP\_Offset$$
2. The burst length causes duplicated retransmission of lost packets (explained in Section 2), the sink node receives multiple copies and the packet marked as received (delete its sequence number from the waiting group); however, only the first copy that has arrived will determine the packet delay value.

The authors in [15] developed a protocol to coordinate network endpoints to measure delay and delay variation (jitter) for IP networks. Based on this protocol, the delay variation $dv_{x}$ is calculated for a sequence of packets $S_{n}$ as the absolute value of subtraction of one-way packet delays $d_{i}$ and $d_{j}$ of two consecutive packets $P_{i}$ and $P_{i+1}$.

(4) $$dv_{x}=\left|d_{i}-d_{j}\right|\;\left(\mathrm{ms}\right),\;i,j\;\in \;S_{n}$$

## 4. Testbed

**Figure 4:**
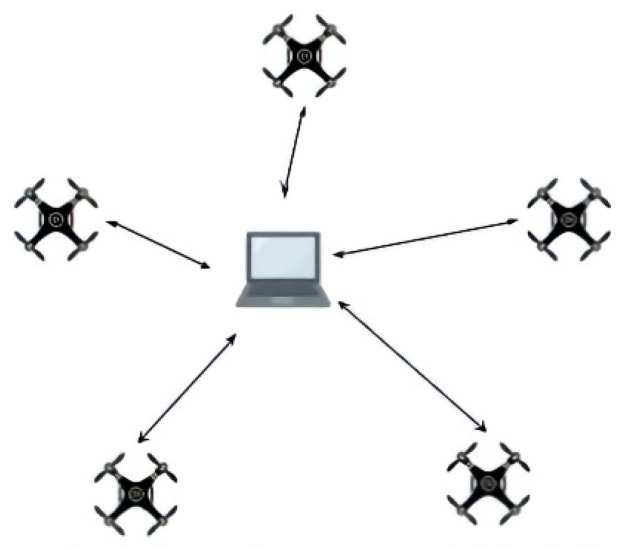
Topology of the experiments.

We conducted experiments to investigate the QoS of MS-AL-ARQ using 5 microcomputers: Raspberry Pi 3 Model B running on the Ubuntu MATE 16.04 operating system as transmitters and an HP laptop running on Ubuntu MATE 16.04 as a control station (destination node). The devices are connected using 802.11 g. We created a standalone wireless network where the nodes were connected in an ad hoc manner (Figure 4).

The MS-AL-ARQ algorithm was implemented on the application layer as a Client/Server Linux-based application in C++. The application is available on GitHub [16]. The client part was installed on the transmitters and the server part was installed on the receiver. Because the application must provide two-way message transfer between a server and a large number of clients without any connection agreement by the server, the best choice to establish data transmission was by using UDP sockets. This choice is also related to the scenario’s requirements, since the delay is crucial and the packet loss will be handled by the application. However, the algorithm can be adapted to work over TCP also.

The application provides a model to generate artificial network packet loss with different values of burst length (LB). This model generates artificial drops on the application layer. This means that some generated packets will not be sent even to the lower layers. To test and evaluate the performances of MS-AL-ARQ, different values of artificial packet loss were set for each experiment. The run commands for both source and receiving node are, respectively, as follows:

*./arqsource -ip 10.0.2.15 -port 5011 -ip_s 10.0.2.1 -port_s 5022 -ploss 0.1 -lb 12 -bsize 2000*

*./arqsink -port_vlc 5014 -bsize 2000 -btime 1000 -tm 1000*

where *-ip*, *-port*, *-ip_s*, *-port_s*, *-ploss*, *-lb*, *-bsize*, *-btime*, and *-tm* are, respectively, the IP address of the sender, sending port, IP address of the receiver, receiving port, artificial packet loss rate (‰), burst length (packets), maximum playback buffer (packets), maximum playback buffer delay (msec), and PLR calculation interval (packets). The authors in [9] explained the relationship between packet loss rate (PLR) metrics and distance in real experiments and showed that PLR ≈ 0.07‰ for UDP transmission indicates the worst-case guarantee value of PLR for one transmission node. Based on these measurements, we set the artificial network packet loss interval for a standard video test sequence and compared it with the measured PLR of the application with MS-AL-ARQ. The values of the LB were artificially generated between the phases of packet formation and packet transmission. Based on the framework model proposed by [17] for short-term loss patterns, we set the average LB to 10 successive packets (probability interval (4; 16)) to give accurate results in packet loss rate calculation and delay measurement.

Experimental parameters are shown in Table 1 The values of the application output parameters are calculated and recorded in log files for each video sequence.

## 5. Results

**Figure 5:**
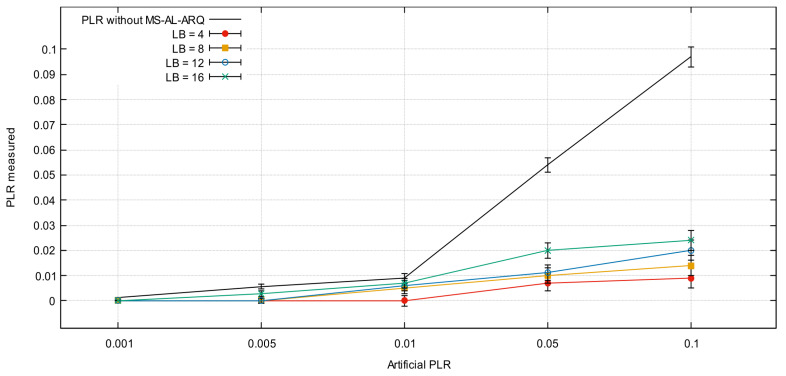
MS-AL-ARQ packet recovery performances for different packet loss rate (PLR) and burst length (LB) values.

Figure 5 represents the performances of MS-AL-ARQ for a single node transmission scenario.

This scenario is used to detect the maximum buffer capacity to handle packet management (in-order delivery, packet request, duplication) under the post-conditional buffer parameters (reordering offset = 200 packets; reordering late time offset = 1 s). Both parameters are used by default in the next experiments.

The reason behind the increase in PLR for each experiment is the variation of burst lost lengths. For each LB, we simulate the packet loss event starting at different frames in the video sequence within a range of 1000 packets [17], and compute the resulting PLR. When LB and PLR are set at the highest value (0.1‰; 16 packets), the application records an average PLR of 0.023. This record is related to the buffer post-condition parameters (reordering bytes offset, reordering late time offset), varying according to the buffer’s parameters. However, changing these parameters is a crucial factor for determining the best QoS. In previous works [9,18], the values (reordering offset = 200 packets, reordering late time offset = 1 s) are defined as the optimal values for peer-to-peer transmission scenarios in real experiments.

### 5.1. MS-AL-ARQ Recovery Delay

**Figure 6:**
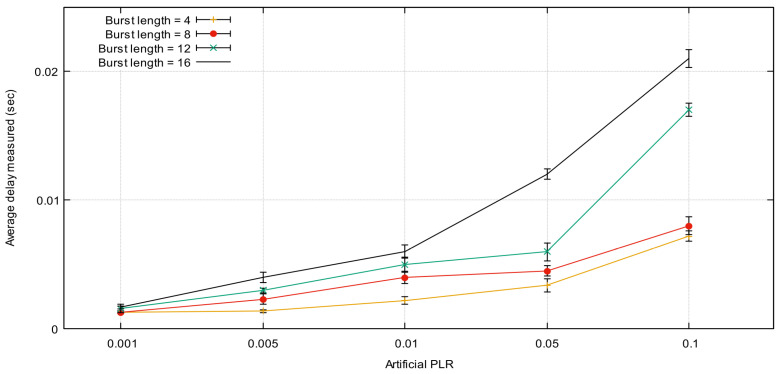
MS-AL-ARQ packet delay (average one-way packet delay) for different packet loss rate (PLR) and burst length (BL) values.

Figure 6 represents the measurement of the average one-way packet delay as a function of PLR for different LB values for 1000 successive data packets. The figure shows an increase in average delay caused by the increase in PLR and LB. This is introduced as a result of requesting and retransmission of the lost packets because the sender needs extra time to detect and resend it. MS-AL-ARQ recorded minimum one-way packet delay (PLR = 0.001) that equals 2 ms. In the worst case of PLR (PLR = 0.1), the maximum average transmission delay equals 21 ms. These values are very acceptable, since MS-AL-ARQ one-way transmission delay needs to go through the application layer, which requires more processing delay.

**Figure 7:**
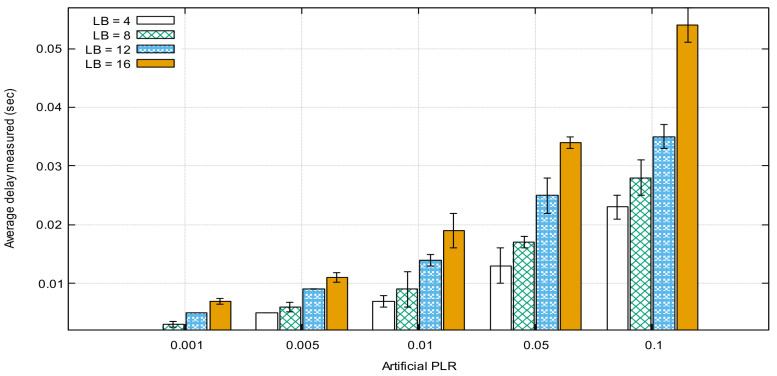
Average delay of retransmitted packets.

Figure 7 represents a comparison between the average delays of only the retransmitted packets for different LB values. Based on these results, an adaptive threshold one-way packet delay could be set depending on the LB and PLR values calculated during the transmission to refine and manage the waiting group buffer, which is used to store the sequence numbers of the requested packets.

**Figure 8:**
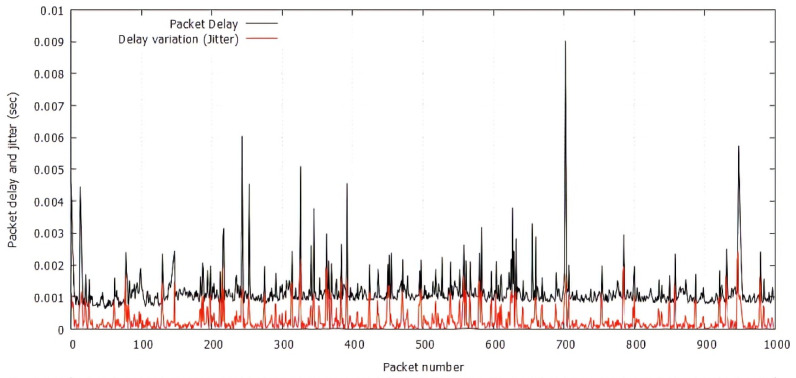
Packet delay and delay jitter for 1000 consecutive received packets.

Figure 8 shows the direct correlation between packet delay and jitter. If the transmission is lossless, the delay and delay jitter do not increase, but if the packet loss event occurs, the delay will be increased by additional recovery time, which leads to an increase in delay variation (jitter). The results also show that jitter of lost packets is spread a little bit more widely, where we recorded a maximum delay jitter (the worst-case delay jitter) up to 2.5 ms, the average jitter was 0.3 ms, and maximum packet delay was up to 9 ms. Note that these results evaluate the performances of the MS-AL-ARQ algorithm in the case of a simple network that does not include any relay nodes. For further research, these metrics will be used to define the optimal values of the parameters of a dynamic jitter buffer on the relay node(s) to control the one-way packet delay between the source and the destination node.

### 5.2. Packet Loss Rate

**Figure 9:**
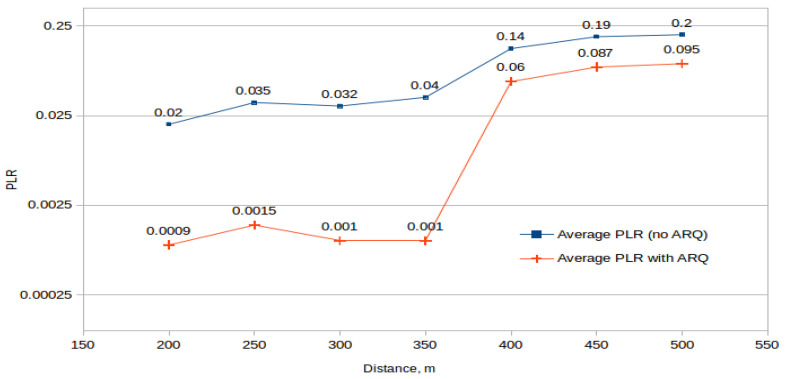
Average packet loss rate (PLR) measured in relation with distance.

We investigate the effectiveness of the proposed algorithm from the point of view of packet recovery and its impact on the transmission delay for a multi-source scenario. During the experiments, artificial packet loss events were generated on the application layer. The generated loss covers all the internal and external influences that may occur because of the special characteristics of the unreliable link and network mobility. For deep investigations related to network interference, especially the network mobility and channel influence, an experimental scenario was proposed where an additional interference was produced due to the flying trajectory of the UAV. This interference disrupted and weakened the Wi-Fi signal. The drone moved straight from the source to the destination and then returned with the same speed. The results of the experiments (measurement of PLR in relation to distance before using the algorithm proposed and while using it) can be viewed in Figure 9. As distance between the source and the destination increases, the algorithm tries to overcome the packet loss. Beginning from the 450 m checkpoint, there was not any chance to recover all lost packets due to the physical limitation of the connection.

**Figure 10:**
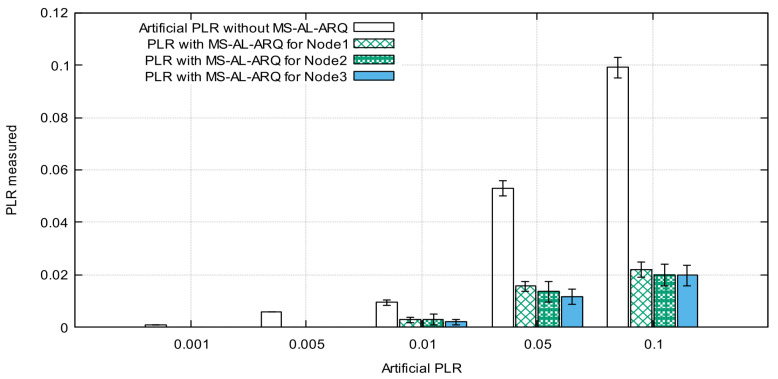
Average packet loss rate (PLR) for 3 nodes.

**Figure 11:**
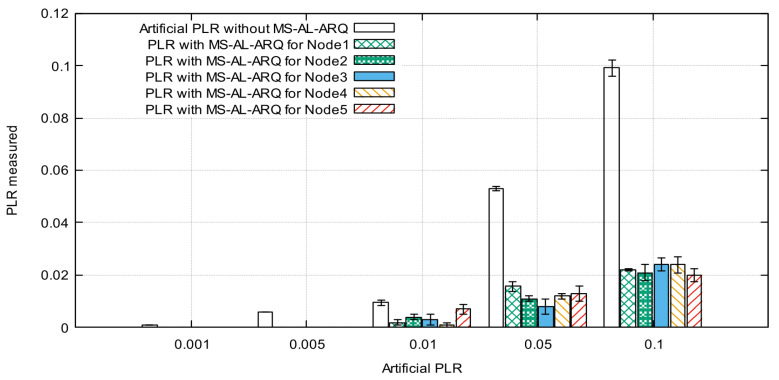
Average packet loss rate (PLR) for 5 nodes.

Figure 10 and Figure 11 represent the average PLR calculated for each node for different parameters values.

The results show a very small difference in PLR values while changing the number of nodes. This is because of the lightweight retransmission mechanism used by the algorithm to request lost packets, which do not force the packet to compete for resources (recovery time and space in buffer). Moreover, the results show that MS-AL-ARQ performs quite well for PLR improvement; we recorded less than 0.022‰ as the highest value for both scenarios. In other words, if 5 nodes are sending data with an average packet loss rate 0.1‰ and burst loss length up to 16 packets for each node, the application could alleviate this loss to 0.02‰ for each node. The results are exclusively due to MS-AL-ARQ’s packet recovery and do not include the performances of other error-control mechanisms on lower layers such as on the data link layer or transport layer in case TCP was used. This is because the packet loss is artificially generated on the application layer, which means that for other layers, the transmission is lossless.

In fact, during the deep analysis of the algorithm’s performances, we have noticed that in some experiments, the algorithm gives a noticeable difference in the PLR calculated for each node, regardless of the experiment conditions matching.

**Figure 12:**
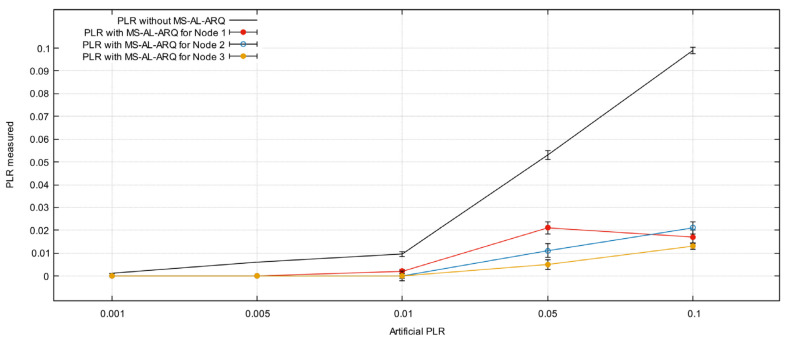
MS-AL-ARQ packet loss rate (PLR) for burst length = 12.

Figure 12 shows the difference in PLR for experiment number 4 (artificial packet loss = 0.05, burst length = 12). The difference is quite noticeable due to the absence of an effective interaction and fairness in the application. The receiver keeps asking for the lost packets by repeating the (NACK) packets steadily until these packets are not relevant for the application or until it receives a cancellation message from the source node. Meanwhile, for each data flow, the loss packet events occur at different frames of the video sequence within the chosen range, which leads to a variation in the number of packets received from each node. Factually, this outstanding absence of fairness requires further research regarding buffer management from the point of packets’ prioritization. This will set up priority filters to process traffic with higher priority before normal traffic in relation to PLR.

### 5.3. Quality of Experience Investigation

**Figure 13:**
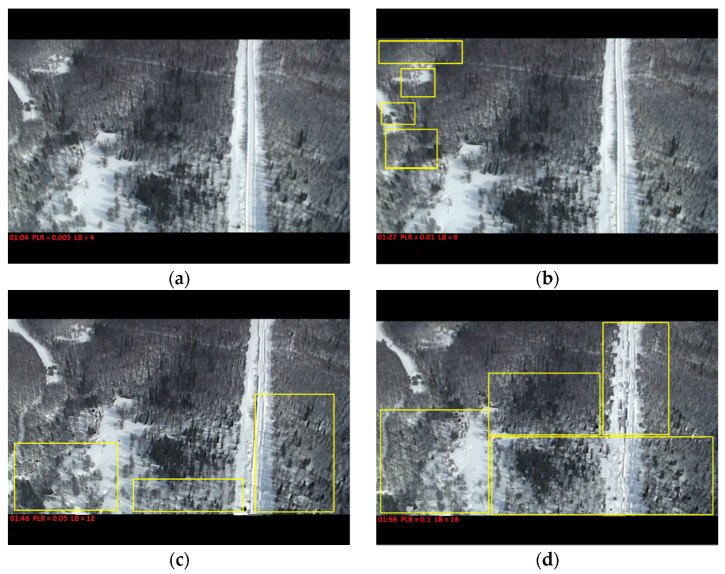
Influence of packet loss rate (PLR) and packet delay on the QoE. (**a**) PLR = 0.005 LB = 4; (**b**) PLR = 0.01 LB = 8; (**c**) PLR = 0.05 LB = 12; (**d**) PLR = 0.1 LB = 16.

Figure 13 represents the influence of PLR and packet delay on the QoE of the video received. It shows the relation between the quality of the video on the destination node and the increase in PLR and LB [19,20]. We used the QoE monitoring system for streaming services proposed by [21], based on the assigned parameters during the experiments (PLR, LB, buffer size, and timeout) and the QoS results of MS-AL-ARQ. We used human observers to rate the received video in terms of Mean Opinion Score (MOS) [22]. The results are presented in Table 2.

The network traffic and application performances will influence the overall quality of the video transmitted [23].

## 6. Conclusions

In this article, we demonstrated an effective and efficient strategy for real-time data transmission over UDP. This strategy is based on a lightweight error control mechanism on the application layer. We calculated and measured recovery delay and PLR as QoS metrics using artificial packet loss and bursts. Moreover, we evaluated the QoE of video data received in terms of mean opinion score (MOS). The results showed that the use of the MS-AL-ARQ algorithm on the application layer for the proposed scenario will alleviate the PLR by retransmitting the lost packets without a strong influence on the average transmission delay. Moreover, it showed that the number of sending nodes will not significantly affect the performance of the MS-AL-ARQ algorithm for a small UAV network (in our case, up to five nodes). However, some buffer congestion issues were detected and set as a catalyst for upcoming research to improve the algorithm from the point of packet prioritization.

## Figures and Tables

**Table 1:** Parameters of experiments.

Parameter	Setting
Operating system	Ubuntu MATE 16.04
Application layer	MS-AL-ARQ
Video coding	H.264
Video resolution	1920 × 1080
Transport layer	UDP
Wireless standard	802.11 g
Video packet size	1.5 Kbyte
NTP offset (ms)	[0.1; 0.8]
Number of sending nodes	[1; 5]
Artificial packet loss rate ‰	[0.001; 0,1]
Artificial burst length	[4; 16]

**Table 2:** Dependence of quality of experience on the packet loss rate (PLR).

Artificial PLR	MOS without MS-AL-ARQ	Quality without MS-AL-ARQ	MS-AL-ARQ PLR	MS-AL-ARQ MOS	Quality with MS-AL-ARQ
0.001	4.96/5	Excellent	0	5/5	Excellent
0.005	4.91/5	Excellent	0	5/5	Excellent
0.01	3.88/5	Fair	0.003	4.46/5	Good
0.05	2.23/5	Poor	0.01	4.34/5	Good
0.1	0.12/5	Very bad	0.023	4.03/5	Fair

## Data Availability

The study did not report any data.
